# Evaluating the Effectiveness of Laparoscopic Removal of an Accessory Spleen After a Failed Splenectomy for Immune Thrombocytopenia

**DOI:** 10.7759/cureus.65876

**Published:** 2024-07-31

**Authors:** Muzi Meng, Paul Joon Koo Choi, Reshma Pydi, Daniel T Farkas

**Affiliations:** 1 School of Medicine, American University of the Caribbean, Cupecoy, SXM; 2 General Surgery, BronxCare Health System, New York, USA; 3 Surgery, BronxCare Health System, New York, USA

**Keywords:** laproscopic splenectomy, accessory spleen, refractory itp, idiopathic thrombocytopenic purpura (itp), immune thrombocytopenia

## Abstract

Immune thrombocytopenic purpura (ITP) is a challenging condition to manage especially when conventional treatment methods, including splenectomy, fail. This report evaluates the effectiveness of laparoscopic removal of accessory spleen for chronic refractory ITP after an initial splenectomy. A 73-year-old African American male with a history of ITP, previously treated with laparoscopic splenectomy nine years ago, presented with severe thrombocytopenia that was found to be refractory to medical therapies. Platelet counts were monitored, and the absence of Howell-Jolly bodies was noted in the peripheral blood smear. Imaging studies over the past eight years indicated the growth of a mass in the left upper abdomen, suggesting a possible accessory spleen. Given the overwhelming evidence of a splenule in refractory thrombocytopenia, laparoscopic exploration and mass removal were conducted. Histologic analysis of the removed mass confirmed a splenule. Despite the complete removal of the mass, postoperative platelet counts remained consistently low and unresponsive to the resumption of medical therapies. This study emphasizes the limitations of accessory splenectomy for refractory ITP and highlights the need for further research to clarify the long-term effectiveness of this surgical procedure in these patients.

## Introduction

An accessory spleen is a splenic mass that develops separately from the normal spleen yet possesses typical splenic characteristics [1]. Clinicians have increasingly considered laparoscopic removal of the accessory spleen as a viable treatment option for patients with immune thrombocytopenic purpura (ITP), a condition characterized by abnormally low platelet counts, which impairs blood clotting during bleeding [1]. This case report presents a review of the literature and an analysis of the utility of accessory splenectomy as a treatment option for ITP patients, particularly when drug medications following a failed splenectomy are not effective or tolerated.

## Case presentation

A 73-year-old African American male with a history of laparoscopic splenectomy for ITP in 2015 was being followed for chronic refractory ITP and failing conservative management. He was on four-week rituximab infusion therapy as an alternative to steroids, which had only provided transient responses and further complicated his diabetes. At his most recent visit to the hematology clinic, he was found to have severe thrombocytopenia of 10,000/μL (Table 1). When the lab results came back, the patient was referred to the emergency room (ER) for planned admission. He was admitted and started on intravenous steroids and intravenous immunoglobulin (IVIG).

**Table 1:** Patient's platelet counts with reference ranges over time

Test	Value	Reference range
Platelet count (first ER visit), Day 1	10,000/μL	150,000-450,000/μL
Platelet count (first discharge), Day 4	283,000/μL	150,000-450,000/μL
Platelet count (returned ER visit), Day 15	10,000/μL	150,000-450,000/μL
Platelet count (subsequent ER visit), Day 38	6,000/μL	150,000-450,000/μL
Platelet count (ICU), Day 58	27,000/μL	150,000-450,000/μL
Platelet count (pre-surgery), Day 64	18,000/μL	150,000-450,000/μL

**Figure 1:**
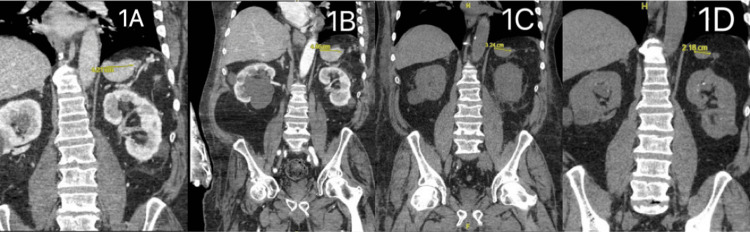
Computed tomography (CT) images display a potential accessory spleen located in the left upper quadrant of the abdomen (A) 08/23 - 4.21 cm, (B) 02/23 - 4.46 cm, (C) 2021 - 3.24 cm, and (D) 2016 - 2.18 cm.

Further review of history and previous workup revealed the absence of Howell-Jolly bodies on the peripheral blood smear, prompting the possibility of an accessory spleen. A computed tomography (CT) scan of the abdomen revealed a mass, possibly an accessory spleen, in the left upper quadrant just below the left hemi-diaphragm. Comparison with prior imaging studies confirmed that the mass had been present since 2016 and gradually increased in size over the years (Figure 1). The patient responded well to medical therapy during this initial admission and was discharged with a platelet count of 283,000/μL, with plans for close follow-up in the clinic (Table 1).

**Figure 2:**
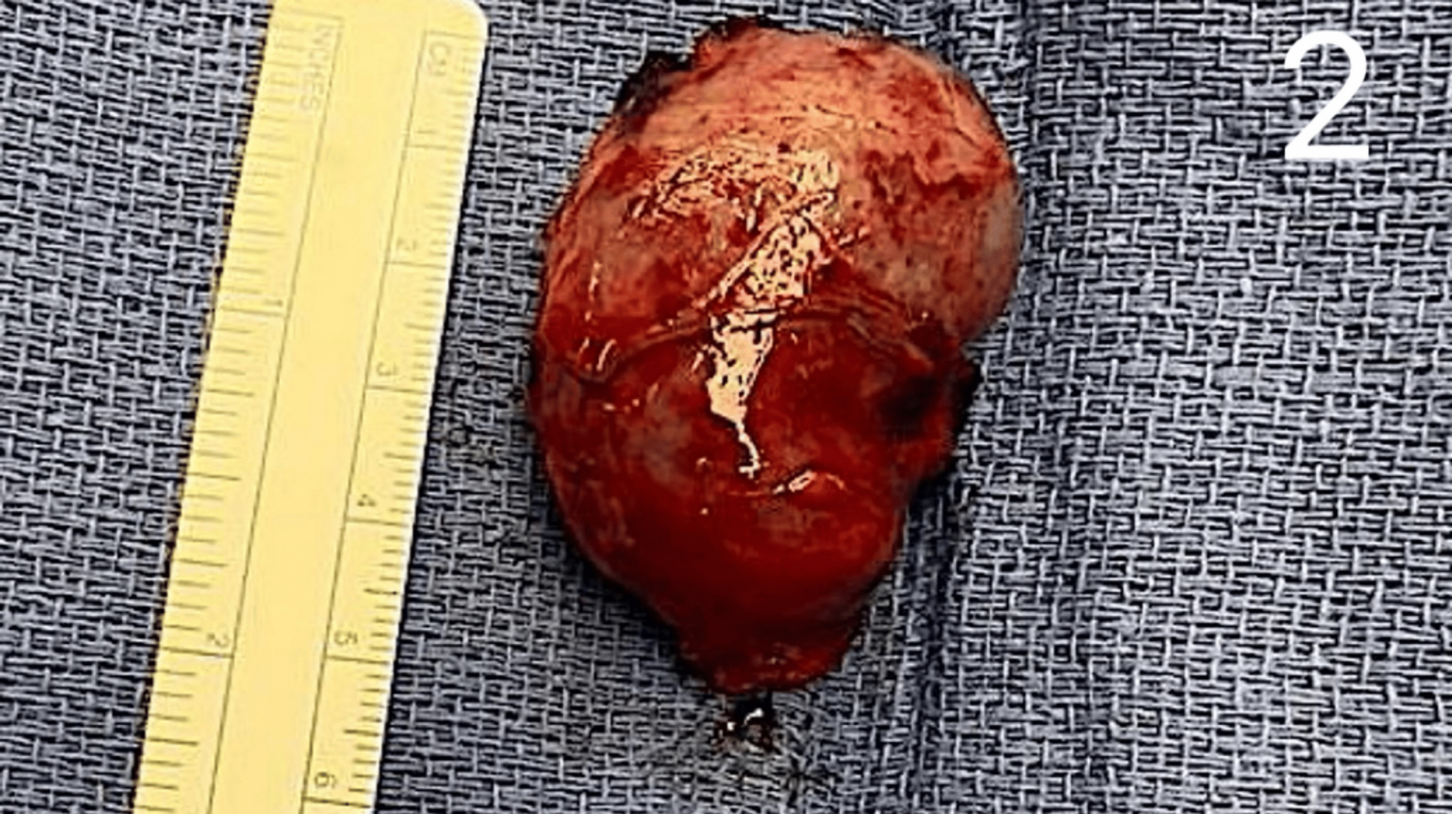
A photograph showing the accessory spleen removed during the operation

The patient returned to the ER two weeks later with a platelet count of 10,000/μL, again devoid of any symptoms. He was discharged following a response to two doses of IVIG, with plans to continue rituximab infusion as an outpatient (Table 1). Three weeks later, he returned once again with a platelet count of 6,000/μL and was admitted to the intensive care unit (ICU) for close monitoring, receiving two additional doses of IVIG (Table 1). After being downgraded to a floor status, he was given intravenous steroids and a platelet transfusion. Subsequently, he received two more doses of IVIG during this admission, resulting in an increase in platelet count to 27,000/μL (Table 1). However, the count subsequently declined to 18,000/μL over the following days, prompting surgical consultation regarding the possibility of accessory splenectomy (Table 1). The patient underwent a repeat laparoscopic splenectomy in October 2023. The omentum was separated from the greater curvature, and the lesser sac was entered. A 3 cm splenule was identified high up toward the diaphragm in the left upper quadrant. A ligature was used to divide the vascular adhesions, and the splenule was removed in a bag (Figure 2).

Postoperatively, the pathological report confirmed splenic tissue. However, there was no immediate improvement in platelet count. The patient underwent Rho(D) immune globulin infusion for refractory ITP but still did not exhibit any improvement in his platelet counts. A nuclear scan was done, which did not show any residual splenic activity. Daily azathioprine therapy was initiated. Although the patient was discharged on postoperative day 36, he was readmitted six months later for persistent hematuria and symptomatic urinary tract infection (UTI). His postoperative blood smear confirmed the presence of Howell-Jolly bodies, indicating complete removal of splenic tissue. The patient had been on 40 mg of prednisone and 100 mg of azathioprine once a day for three months. He is currently on 40 mg of prednisone and 150 mg of azathioprine daily.

## Discussion

Immune thrombocytopenic purpura

ITP is an autoimmune condition characterized by abnormally low platelet levels due to autoantibodies that attack and destroy platelets [1]. The spleen, containing antigen-presenting macrophages, plays a significant role in the clearance of platelets, contributing to their reduced numbers [1]. Consequently, the bone marrow attempts to compensate by increasing platelet production, although this often fails to correct the platelet deficiency in ITP [2]. Typically, individuals with ITP have platelet counts below 100,000 per microliter (µL), significantly lower than the normal range of 150,000 to 400,000/µL [2]. ITP manifests when platelet counts drop without a clear underlying cause [2].

Chaturvedi et al. emphasized the spleen's important role in ITP pathogenesis, highlighting its function as the primary site for platelet clearance and the creation of immune cells that foster anti-platelet antibody production [3]. Consequently, splenectomy, the surgical removal of the spleen, is a known treatment for ITP. By eliminating the tissue responsible for platelet clearance and autoantibody production, splenectomy allows ITP patients to maintain platelet levels. Chaturvedi et al. reported that 80% of ITP patients undergoing splenectomy experience an immediate increase in platelet count, underscoring the procedure's effectiveness in ITP management [3].

ITP patients often present with purpura from the extravasation of blood from capillaries into the skin [2]. This clinical manifestation of unexplained bruises may serve as an early sign of ITP. In children, purpura associated with ITP typically appears within a few weeks following a viral infection, indicating a potential cross-immunization between viral and platelet antigens [2]. When the platelet count falls below 50,000/µL, bleeding tends to ensue, and intracranial hemorrhage can occur if the count drops below 10,000/µL [2].

The role of splenectomy in immune thrombocytopenic purpura

Splenectomy remains a popular second-line treatment for ITP. However, it carries inherent surgical risks such as bleeding and infection. Therefore, the decision to proceed with splenectomy should carefully consider individual patient factors including age, concurrent health conditions, disease duration, response to prior therapies, and predictive indicators of treatment success [4]. Splenectomy is indicated if initial therapies such as steroids, intravenous immunoglobulin (IVIG), and anti-D immunoglobulin prove ineffective [4]. Additional indications include young patients without comorbidities, patients with persistent or chronic ITP that was diagnosed more than 12 months ago, and those without severe bleeding [5]. For instance, Rijcken et al. identified younger age at the time of surgery, higher platelet counts before splenectomy, and shorter duration of ITP to be associated with better long-term outcomes [6]. Moreover, patients who responded well to corticosteroids or IVIG before splenectomy were more likely to sustain that response after surgery [6].

The British Pediatric Hematology Group and the American Society of Hematology endorse splenectomy as a preferred second-line therapy for ITP [5]. Their guidelines advise delaying splenectomy in children until they have had ITP for at least 12 months and caution against performing the procedure in those with active bleeding symptoms [5]. This approach aims to minimize the risk of severe bleeding complications post-surgery, which can be particularly critical in pediatric patients. In certain cases, non-surgical treatments like thrombopoietin receptor agonist (TPO-RA) may offer a more suitable alternative due to its lower risk profile, though TPO-RA success often requires long-term treatment, posing challenges associated with treatment adherence [4].

Evaluating residual splenic function after unsuccessful splenectomy

When primary splenectomy fails to achieve desired outcomes in patients with ITP, assessing residual splenic tissue becomes crucial. Accessory spleens can perpetuate platelet clearance and antibody production, maintaining the disease process. The initial step in evaluating residual splenic function involves examining the peripheral blood smear for Howell-Jolly bodies, which are nuclear remnants typically removed by functioning splenic tissue in the body [7]. The presence of Howell-Jolly bodies indicates the absence of functional splenic tissue post-splenectomy. Conversely, their absence suggests the possibility of residual splenic activity.

If the peripheral smear is inconclusive, imaging studies become essential. Contrast-enhanced CT scans are highly sensitive in detecting splenic tissue and providing detailed anatomical information as seen in our case [8]. MRI offers effective soft tissue contrast without ionizing radiation but may be limited by accessibility and cost. Another method with a good sensitivity profile is technetium-99m-labeled heat-damaged red blood cell scintigraphy (99mTc-HDRBC), which visualizes splenic remnants by concealing heat-damaged red blood cells [9].

Altaf et al. reviewed seven patients who underwent laparoscopic accessory splenectomy for recurrent ITP after a failed initial splenectomy [10]. Five of the seven (71.4%) had sustained improvement in platelet counts [10]. The authors emphasized the importance of perioperative localization methods, such as preoperative CT-guided methylene blue injection and 99mTc-HDRBC, in aiding the intraoperative identification of accessory spleens, especially for unusual locations [10].

Accessory spleen in immune thrombocytopenic purpura

Accessory spleens are reported to be found in 10%-30% of the general population, with approximately 17% located within the tail of the pancreas [11]. The presence of accessory splenic tissue can significantly impact the management and outcomes of patients with ITP, particularly those who have already undergone splenectomy. Several studies emphasized the importance of identifying and removing accessory spleens in those with refractory ITP. Altaf et al. reported that five out of seven patients (71.4%) who underwent laparoscopic accessory splenectomy for recurrent ITP after initial failed splenectomy showed continued improvement in platelet counts [10]. They emphasized the significance of perioperative localization methods to help with intraoperative identification of accessory spleens [10].

Failure to remove accessory splenic tissue during initial splenectomy can lead to persistent or recurrent thrombocytopenia, as seen in our presented case, and may result in treatment failure [7]. Therefore, preoperative imaging and thorough intraoperative exploration to identify and remove all splenic tissue are crucial. AlShammari et al. reported a case, in which a laparoscopic removal of an intrapancreatic accessory spleen resulted in sustained improvement in platelet counts and allowed for the tapering of steroid therapy [11].

Preventing splenic tissue spillage during laparoscopic splenectomy is also a topic of discussion. Spillage during the initial surgery can lead to the development and growth of an accessory spleen. During the initial splenectomy in our presented case, the patient’s 2015 operative report mentioned possible spillage of splenic tissue during morcellation from a large hole in the Endo Catch™. We speculate this may have led to the development of the accessory spleen. Similarly, Lansdale et al. presented a case of recurrent ITP in a patient who received laparoscopic splenectomy complicated by rupture of the retrieval bag, leading to intra-abdominal splenosis [7]. Lansdale et al. recommended using blunt instruments for morcellation to reduce the risk of bag rupture [7]. If splenic tissue spillage occurs during the procedure, meticulous inspection, suction, and lavage are necessary as even small amounts of residual splenic tissue may lead to splenosis and ITP recurrence, potentially negating the benefits of the initial surgery [7]. Alternative retrieval methods may be considered for larger spleens to reduce the risk of tissue spillage. Altaf et al. suggested techniques such as a Pfannenstiel retrieval incision or hand-assisted laparoscopic approach as safe alternatives to morcellation for large spleens [10]. These methods provide better control and visibility during the removal process, reducing the likelihood of accidental tissue spillage and seeding.

The role of accessory splenectomy in refractory immune thrombocytopenic purpura

Accessory splenectomy has become a critical intervention for patients with refractory ITP who experience disease recurrence following primary splenectomy. By removing residual splenic tissue, accessory splenectomy may enhance long-term outcomes and reduce the necessity for ongoing immunosuppressive therapy.

Rudowski et al. identified an accessory spleen in 110 (18%) of 611 patients who underwent splenectomy for hematological diseases [12]. Among 177 patients who underwent splenectomy for ITP, 52 experienced recurrent thrombocytopenia, with 19 cases showing long-term recurrence. Accessory spleens were confirmed via scans and during re-operation in these patients [12]. Following the removal of the accessory tissue, thrombocytopenia resolved in 12 of the 19 patients [12]. This study underscores the importance of detecting and excising accessory spleens during initial splenectomy to prevent ITP recurrence. The availability of minimally invasive laparoscopic techniques has rendered accessory splenectomy an increasingly favorable option for managing these complex cases, potentially enhancing long-term outcomes.

Choi et al. reported a 44-year-old male with ITP who relapsed after splenectomy and remained unresponsive to various treatments for over 20 years [13]. Imaging studies showed an accessory spleen, prompting laparoscopic accessory splenectomy [13]. Although the patient initially experienced an increase in platelet counts after surgery, subsequent reductions occurred. Nonetheless, lower corticosteroid doses were required to maintain platelet levels compared to pre-surgery, indicating potential benefit in mitigating disease severity for refractory ITP patients with identified accessory splenic tissue [13]. They were in support of surgical removal of residual accessory splenic tissue in those with recurrent ITP after the initial splenectomy and noted that splenectomy has 50%-70% long-term effectiveness and that patients relapse because of accessory splenic tissue [13]. They also emphasized that the availability of laparoscopic techniques, which are safe and less morbid, may render surgeons’ decision to operate easier [13].

Costa-Pinho et al. retrospectively analyzed data from 53 patients who underwent laparoscopic splenectomy for the treatment of chronic ITP [9]. They showed that 94.3% achieved a treatment response [9]. During the mean follow-up of 24.8 months, 75.5% of their patients maintained a complete response, and an additional 22.4% had platelet counts consistently above 30,000/µL. About 79.6% of patients did not require more treatment after surgery [9]. They confirmed the safety and efficacy of laparoscopic splenectomy as a second-line treatment for ITP, with satisfactory short-term and long-term outcomes [9].

Montalvo et al. studied 150 patients with laparoscopic splenectomy for refractory ITP [8]. The patients were observed for one year after surgery, and the results showed an 88.7% complete response rate, a general 2.7% rate, and an overall effectiveness rate of 91.4%. In addition, the results showed that the patients who achieved ≥150,000 platelet counts within one week after surgery proved to have a high complete rate of 94.2%. Despite the above results, there was still a need for effective and controlled research endeavors to identify the accurate indicators that can predict surgical effectiveness.

Effectiveness of accessory splenectomy in refractory ITP

Despite the above-mentioned effectiveness of the procedure, accessory splenectomy in the management of refractory ITP remains controversial. While some studies report sustained improvement in platelet counts following the removal of residual splenic tissue, others suggest that the procedure may not always lead to complete remission.

Riaz et al. presented a rare case of a 30-year-old female with refractory ITP who underwent accessory spleen removal seven years after her initial splenectomy [14]. Despite a temporary increase in platelet count postoperatively, the patient had recurrent attacks of thrombocytopenia and eventually passed away, suggesting that accessory spleen removal may not always be effective. In addition, Leo et al. reported two patients who underwent laparoscopic accessory splenectomy for recurrent ITP [15]. While both patients had a transient disease-free period (two months and one month, respectively), they ultimately required a resumption of immunosuppressive therapy, indicating that surgical accessory splenectomy may only allow for temporary remission [16]. Szold et al. followed eight patients who underwent laparoscopic accessory splenectomy [16]. None of their patients achieved complete remission, with two having partial remission and five requiring chronic corticosteroids to maintain a platelet count [16].

The effectiveness of accessory splenectomy in refractory ITP varies among patients. While some studies report sustained improvement in platelet counts and reduced need for medical therapy, others suggest that the procedure may only provide temporary remission or fail to achieve complete remission. Despite the inconsistent outcomes, the availability of a safe, minimally invasive laparoscopic approach makes the decision to operate more feasible, especially when considering the potential benefits for patients with limited treatment options. Careful patient selection and thorough preoperative imaging to identify accessory splenic tissue are crucial in optimizing outcomes.

Laparoscopic approach of accessory splenectomy

The laparoscopic approach to accessory splenectomy has become increasingly favored due to its minimally invasive nature, which offers several advantages over the traditional open technique. Many studies, including those discussed above, have demonstrated the efficacy and safety of laparoscopic removal, even in patients with complex cases of refractory ITP.

Altaf et al., AlShammari et al., and Choi et al. utilized laparoscopic techniques in their respective studies to address an accessory spleen and reported that patients tolerated the procedures well and experienced various degrees of improvement in their platelet counts [10,11,13]. These studies underscore the benefits of the laparoscopic approach, including reduced postoperative pain, shorter hospital stays, faster recovery times, and smaller incisions, which lead to better cosmetic outcomes. Szold et al. and Morris et al. also reinforced that laparoscopic accessory splenectomy is a well-tolerated procedure with a good safety profile [16,17]. Patients in these studies tolerated the procedure well, demonstrating that this method was practical and effective, even when complete remission was not achieved. Lansdale et al. and Kaban et al. showed that using advanced techniques and devices, such as hand-assist devices and blunt instruments for morcellation, can further enhance safety and effectiveness [7,18]. These methods help prevent complications like splenic tissue spillage and subsequent splenosis.

In summary, the laparoscopic approach to accessory splenectomy offers significant benefits, including less invasiveness and quicker recovery, making it a suitable option for many patients, including those with challenging clinical presentations. The successful management of patients in the referenced studies supports the use of laparoscopic techniques for accessory splenectomy, highlighting their effectiveness and safety in the treatment of ITP.

## Conclusions

Accessory spleens can significantly contribute to the failure of primary splenectomy in patients with refractory ITP, leading to continued thrombocytopenia and necessitating additional surgical interventions. Laparoscopic accessory splenectomy is a feasible and minimally invasive option for addressing this issue. However, this case report and the accompanying literature review demonstrate that there are mixed results regarding its success rate, with overall chances of success being lower than those seen with primary splenectomy. While there are limited reports of laparoscopic accessory splenectomy, our case report demonstrates that it can be performed safely, contributing to the growing body of evidence supporting this minimally invasive approach. Further research is warranted to identify factors that can predict a successful outcome after accessory splenectomy in recurrent ITP.
